# Formation and Coloring Mechanism of Typical Aluminosilicate Clay Minerals for CoAl_2_O_4_ Hybrid Pigment Preparation

**DOI:** 10.3389/fchem.2018.00125

**Published:** 2018-04-19

**Authors:** Anjie Zhang, Bin Mu, Xiaowen Wang, Lixin Wen, Aiqin Wang

**Affiliations:** ^1^Key Laboratory of Clay Mineral Applied Research of Gansu Province, Center of Eco-material and Green Chemistry, Lanzhou Institute of Chemical Physics, Chinese Academy of Sciences, Lanzhou, China; ^2^University of Chinese Academy of Sciences, Beijing, China; ^3^Northwest Yongxin Coatings Limited Company, Lanzhou, China

**Keywords:** CoAl_2_O_4_, aluminosilicate minerals, hybrid pigments, formation mechanism, coloring mechanism

## Abstract

Different kinds of aluminosilicate minerals were employed to fabricate CoAl_2_O_4_ hybrid pigment for studying its formation and coloring mechanism. It revealed that the color of the obtained hybrid pigments was determined by the content of Al_2_O_3_ and lightness of clay minerals. The higher the Al_2_O_3_ content and the lightness of clay minerals, the better the color parameters of hybrid pigments. During the preparation of hybrid pigments, CoAl_2_O_4_ nanoparticles were confined to be loaded on the surface of the aluminosilicate minerals, which effectively prevented from the aggregation and the size increase of CoAl_2_O_4_ nanoparticles. What's more, aluminosilicate mineral might be an ideal natural aluminum source to compensate the aluminum loss due to the dissolution of Al(OH)_3_ at alkaline medium during precursor preparation, keeping an optimum molar ratio of Co^2+^/Al^3+^ for formation of spinel CoAl_2_O_4_ pigments in the process of high-temperature crystallization.

## Introduction

AB_2_O_4_ spinel complex oxides usually are used as ceramic materials, inorganic pigments, magnetic materials, catalysts and gas-sensitive materials (Ren et al., 2014; Yoneda et al., 2016; Zou and Zheng, 2016; Álvarez-Docio et al., 2017; Chafi et al., 2017; Rani, 2017; Tang et al., 2017), the most famous one of them is cobalt aluminate (CoAl_2_O_4_). As a high-grade eco-friendly intense blue pigment, it can be applied in the fields of ceramics, plastics, paint, glass, and color TV tubes due to high refractive index, excellent chemical, and thermal stability (Mahé et al., 2008; Ryu et al., 2008; Tirsoaga et al., 2011; Merino et al., 2015; Soleimani-Gorgania et al., 2015). However, the high cost of CoAl_2_O_4_ pigments has severely restrained their wide applications because of the expensive cobalt compounds (De Souza et al., 2009; Gholizadeh and Malekzadeh, 2017; Zhang et al., 2017). In addition, the traditional method for preparation of cobalt blue pigment was involved in the calcination of CoO and Al_2_O_3_ at above 1,300°C for a long time (Armijo, 1969; Salavati-Niasari et al., 2009; Sale, 2015; He et al., 2017; Zhang et al., 2017), which was obviously time-consuming. Therefore, it is urgent to develop a strategy to prepare the low-cost cobalt blue pigment with prefect color property in order to realize its wide applications.

Incorporation of non-toxic and low-cost elements may be an efficient method to decrease the use of Co element. Torkian et al. synthesized Co_x_Mg_1−x_Al_2_O_4_ nanopigment based on the substitution of Co^2+^ using Mg^2+^ via combustion method (Torkian et al., 2013). Khattab et al. also synthesized Co_x_Mg_1−x_Al_2_O_4_ blue pigment after replacing of Co^2+^ with Mg^2+^ during the calcination process (Khattab et al., 2017), and Sedghi prepared Co_x_Zn_1−x_Al_2_O_4_ nano-pigments by gel combustion method (Sedghi et al., 2014). It suggests that the doping technology using the matched metal ions can enhance the color properties as well as decrease the cost of cobalt blue. However, the decrease in the cost of cobalt blue is limited by the substitution of Co^2+^ using other metal ions. What's more, the agglomeration and crystal grain growth of cobalt blue nanoparticles still remain during calcining process.

Recently, many inorganic substrates are employed to construct the eco-friendly high-grade inorganic hybrid pigment (Mousavand et al., 2006; Zhang et al., 2015; Meng et al., 2016; Mishra et al., 2017; Tian et al., 2017). Due to the abundance in nature, low-cost, non-toxic and unique structure features, clay minerals can be served as a promising substrate for loading of the inorganic nanoparticles (Wang et al., 2011; Todorova et al., 2014; Mu and Wang, 2015; Ezzatahmadi et al., 2017; Intachai et al., 2017). Therefore, our groups have prepared cobalt blue hybrid pigments after incorporating of attapulgite, halloysite (Hal), montmorillonite (Mt), etc., via co-precipitation method followed by a calcination process (Mu et al., 2015; Zhang et al., 2017). It has confirmed that the introduction of clay minerals dramatically decreases the cost of pigment, as well as improves the aggregation of cobalt blue nanoparticles. In addition, some components of clay minerals might enter into tetrahedral or octahedral positions of CoAl_2_O_4_ spinel structure to substitute Co^2+^ or Al^3+^, which have an obvious effect on the color properties of cobalt blue. However, the relevant formation and coloring mechanism of the CoAl_2_O_4_/clay mineral hybrid pigments was still not clear. Therefore, several of typical aluminosilicate minerals were selected to construct the CoAl_2_O_4_/aluminosilicate clay mineral hybrid pigments to study its relevant formation and coloring mechanism in this study, the involved clay minerals included Hal, Mt, kaoline (Kaol), andalusite (And), dickite (Dic), mullite (M47 and M70, the number is indicator of the Al_2_O_3_ content). The effect of the different aluminosilicate clay minerals on the color parameters of hybrid pigment was studied in detail, and the possible formation and coloring mechanism of the hybrid pigments was proposed. It is expected to provide guidance for preparation of low-cost and high-grade cobalt blue with the prefect color properties.

## Experimental

### Materials

Hal and And were obtained from Zhengzhou Jinyangguang Ceramics Co., Ltd. (HeNan, China). Mt, Dic, M47 and M70 were obtained from Yixian Kaolin Development Co., Ltd. (HeBei, China), Qingdao Yuzhou chemical Co., Ltd. (ShanDong, China), Huakang Non-Metallic Minerals Processing Plants (HeBei, China), respectively. Kaol was purchased from Longyan Kaolin Co., Ltd. (FuJian, China). In order to analyze the compositions of aluminosilicate minerals using X-ray fluorescence, the aluminosilicate minerals were firstly crushed and purified by 4% HCl (wt%) to remove carbonates, and then the purified clay minerals were filtered by passing through a 200-mesh sieve. The composition of the involved aluminosilicate minerals is summarized in Table 1. Co(NO_3_)_2_·6H_2_O and Al(NO_3_)_3_·9H_2_O were purchased from Shanghai Reagent Factory (Shanghai, China). NaOH, HCl, and anhydrous ethanol were obtained by China National Medicines Co., Ltd.

**Table 1:** Chemical composition of different clay minerals after acid treatment.

**Clay minerals**	**Al_2_O_3_ (%)**	**Na_2_O (%)**	**MgO (%)**	**CaO (%)**	**SiO_2_ (%)**	**K_2_O (%)**	**Fe_2_O_3_ (%)**	**TiO_2_ (%)**
Hal	29.49	0	0.39	0.08	41.15	0.61	1.71	–
Kaol	54.2	0.014	0.45	0.32	23.5	3.3	0.67	1.36
Mt	22	–	–	–	64.6	5.24	6.03	1.34
Dic	26.2	–	–	0.24	54.1	0.49	0.26	–
And	56.5	–	–	0.37	38.7	1.18	1.39	1.38
M47	49.1	–	–	0.92	42.8	0.96	2.38	3.07
M70	64.6	–	–	0.99	25.5	1.17	2.1	1.02

### Preparation of CoAl_2_O_4_/aluminosilicate clay mineral hybrid pigment

The CoAl_2_O_4_/aluminosilicate clay mineral hybrid pigments were fabricated according to the similar procedure reported in our previous study (Zhang et al., 2017). Co(NO_3_)_2_·6H_2_O (0.01 mol), Al(NO_3_)_3_·9H_2_O (0.02 mol), and 1.090 g of aluminosilicate clay mineral (60 wt% of CoAl_2_O_4_) were added into water (50 mL) and magnetically stirred at 150 rpm for 1 h. And then the pH value of the reaction system was adjusted to 10 using 3 M NaOH aqueous solution and stirred for 2 h at room temperature. The obtained solid products were collected by centrifugation, and washed more than five times using water before being dried at 60°C for 10 h. Finally, it was calcined at 1,100°C for 2 h with a rate of 10°C/min from room temperature to 1,100°C, and the as-prepared hybrid pigments were abbreviated to Kaol-HP, Hal-HP, Mt-HP, M47-HP, M70-HP, And-HP, and Dic-HP corresponding to different clay minerals, respectively. As a control, cobalt blue pigments without clay minerals also were fabricated by the same procedures, and the productions were labeled to CoAl_2_O_4_-900, CoAl_2_O_4_-1000, CoAl_2_O_4_-1100, and CoAl_2_O_4_-1200 according to the calcining temperatures, respectively. In additional, different clay minerals were also calcined at 1,100°C and defined as Kaol-1100, Hal-1100, Mt-1100, M47-1100, M70-1100 And-1100, and Dic-1100, respectively.

### Characterization

The morphology was measured using transmission electron microscopy (TEM, JEM-1200EX/S, JEOL). The structure and composition was analyzed using Fourier Transform infrared (FTIR, Thermo Nicolet NEXUS TM, Madison, USA). The X'pert PRO diffractometer (λ = 1.54060Å) was used to analysis the XRD patterns of the sample with a scan step size of 0.02° per second. Raman spectra were recorded using the microprobe on a Labram HR Evolution Raman spectrometer (Horiba). The Color-Eye automatic differential colorimeter (X-Rite, Ci 7800) was used to study the color properties of the as-prepared pigments by the Commission Internationale de l'Eclairage (CIE) 1976 L^*^, a^*^, b^*^ colorimetric method. L^*^ is the lightness axis (0 for black and 100 for white). The parameters of a^*^ (negative values for green and positive values for red) and b^*^ (negative values for blue and positive values for yellow) denote the hue or color dimensions.

## Results and discussion

### Characterization of Kaol-HP

Figure S1 (see ESI) gives the CIE parameters of Kaol-HP calcined at different temperatures. It is observed that the L^*^ value of hybrid pigments firstly increases with the increase of the calcining temperatures, and then it decreases as the temperature is above 1,100°C. The same change trend is also observed from the value of b^*^ of Kaol-HP, but the color of CoAl_2_O_4_ pigments without Kaol is different compared with that of Kaol-HP prepared under the same conditions. In order to obtain the blue color, the calcining temperature for preparation of CoAl_2_O_4_ pigments without Kaol must be above 1,200°C (Figure S2, see ESI), but its color properties (L^*^ = 37.41, a^*^ = −0.52, b^*^ = −41.14) are poor compared with Kaol-HP prepared at 1,100°C (L^*^ = 48.11, a^*^ = 2.64, b^*^ = −63.75). Thus these two samples are selected to investigate the effect of introduction of clay minerals on the structure and properties of pigments. However, the L^*^ value of Kaol-HP decreases when the temperature increases to 1,200°C, which might be due to the crystal phase transition and the collapse of Kaol structure (Juneja et al., 2010; Yeo, 2011; Zhang et al., 2017).

**Figure 1:**
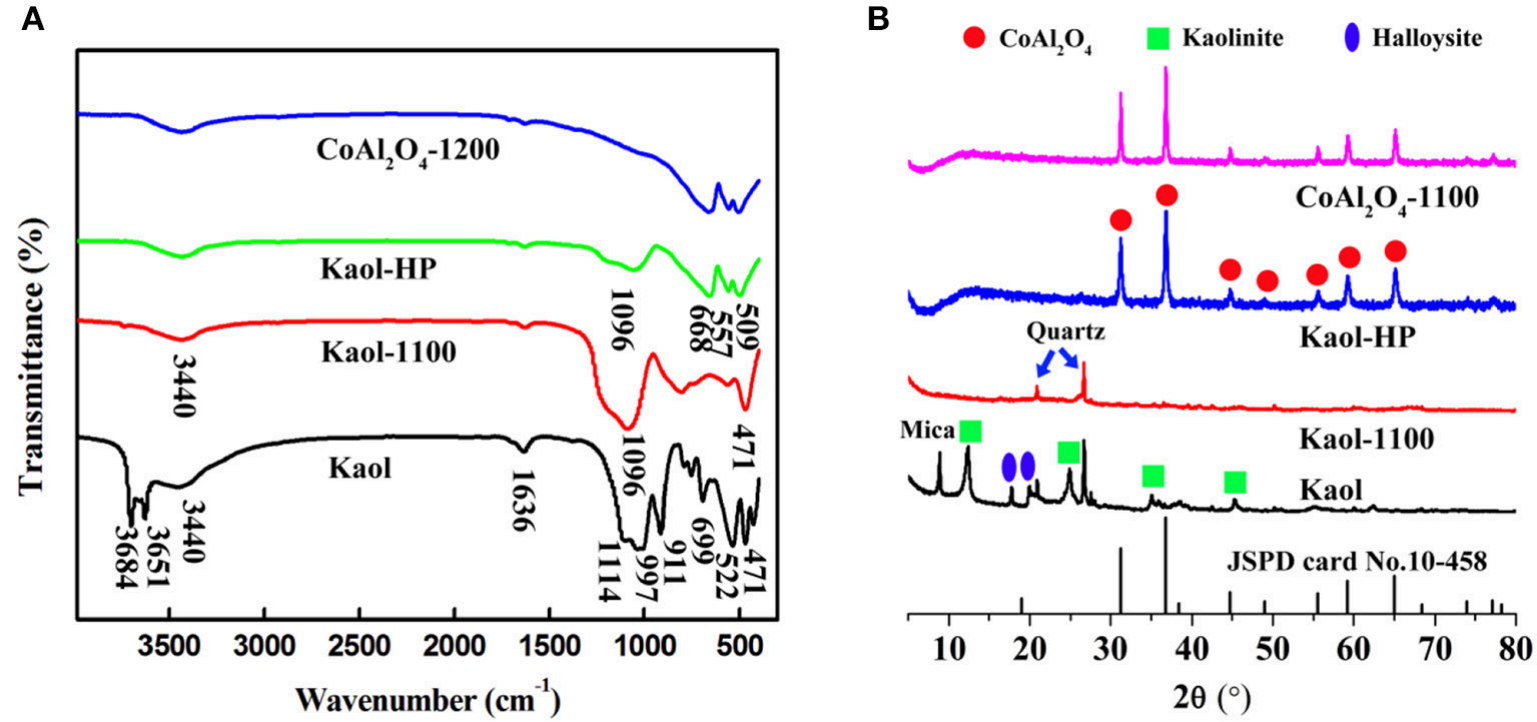
**(A)** FTIR spectra and **(B)** XRD patterns of the raw Kaol, Kaol-1100, Kaol-HP, and CoAl_2_O_4_-1200, respectively.

Figure 1A presents the FTIR spectra of the raw Kaol, Kaol calcined at 1,100°C, Kaol-HP, and CoAl_2_O_4_ calcined at 1,200°C. As depicted in the FTIR spectrum of the raw Kaol, the band at 3,684 and 3,651 cm^−1^ are assigned to stretching vibrations of Al-OH (Saikia and Parthasarathy, 2010). The bands at 3,440 and 1,636 cm^−1^ are assigned to the physisorbed water on the surface of Kaol and the bending vibration of H-O-H, respectively. The IR peaks at 911 cm^−1^ can be ascribed to the Al-Al-OH vibration of the clay sheet, while the bands located at 1,114, 1,096, 1,032, and 471 cm^−1^ are related to Si-O-Si of the clay tetrahedron sheets (Jafari and Hassanzadeh-Tabrizi, 2014). After being calcined at 1,100°C, the typical absorption bands of Kaol at 3,651, 3,684, and 911 cm^−1^ disappear due to the dehydroxylation of Kaol during calcination. After incorporating of CoAl_2_O_4_ nanoparticles, the characteristic absorption bands of CoAl_2_O_4_ at 668, 557, and 509 cm^−1^ were clearly observed, which correspond to the stretching vibration of Al-O of AlO_6_ and Co-O of CoO_4_ (Chapskaya et al., 2005), respectively. Furthermore, these characteristic adsorption bands also can be found in the FTIR spectrum of CoAl_2_O_4_ pigments.

Figure 1B exhibits the XRD patterns of the raw Kaol, Kaol calcined at 1,100°C, Kaol-HP, and CoAl_2_O_4_ calcined at 1,200°C. The raw Kaol presents well-defined reflections at 2θ = 12 and 25°, which are typical characteristic peaks of kaolinite (Panda et al., 2010; Konduri and Fatehi, 2017). The diffraction peak of mica is observed at *2*θ = 8.9° while the ones located at *2*θ = 18.3 and 20.2° are attributed to the diffraction peaks of Hal. It indicates that the raw Kaol is associated with Hal, quartz and mica. When Kaol is calcined at 1,100°C, the typical diffraction peaks of Kaol, Hal, and mica disappear, and only the diffraction peaks of quartz remain. As for Kaol-HP, it is obvious that the typical diffraction peaks of Kaol vanish accompanied with the presence of some new diffraction peaks, which are assigned to spinel CoAl_2_O_4_. According to JCPD card No. 10-458, the diffraction peaks located at *2*θ = 31.1, 36.8, 44.8, 49.0, 55.5, 59.2, 65.2° correspond to (220), (311), (400), (331), (422), (511), and (440) of CoAl_2_O_4_, respectively (Abaide et al., 2015). In order to study the effect of calcining temperatures and incorporating of clay minerals on the sizes of CoAl_2_O_4_, the crystallite size is also calculated using Sherrer relation according to XRD patterns of all samples (including hybrid pigments prepared using Kaol at different temperatures, see Figure S3) (Equation 1):

(1) $$\begin{array}{r} D=\frac{0.89\times \lambda }{B\times \text{cos }\theta } \end{array}$$

where *D* is the crystallite size, λ is the wavelength (Cu Ka), θ is the diffraction angle of the most intense diffraction peak (Zhang et al., 2018), *B* is the corrected half-width obtained using a quartz as reference. *B* = *B*_*m*_–*B*_*s*_, where *B*_*m*_ refers to the tested results and *B*_*s*_ was got by testing a reference substance. As shown in Table S1, it is clear that the crystallite sizes of CoAl_2_O_4_ increases with the increase of the calcining temperatures either hybrid pigments or CoAl_2_O_4_ pigments without Kaol. However, the crystallite sizes of the as-prepared hybrid pigments are smaller than that of CoAl_2_O_4_ pigments, suggesting that the introduction of clay minerals obviously prevents from the increase in size and the agglomeration of CoAl_2_O_4_ particles during calcining process.

**Figure 2:**
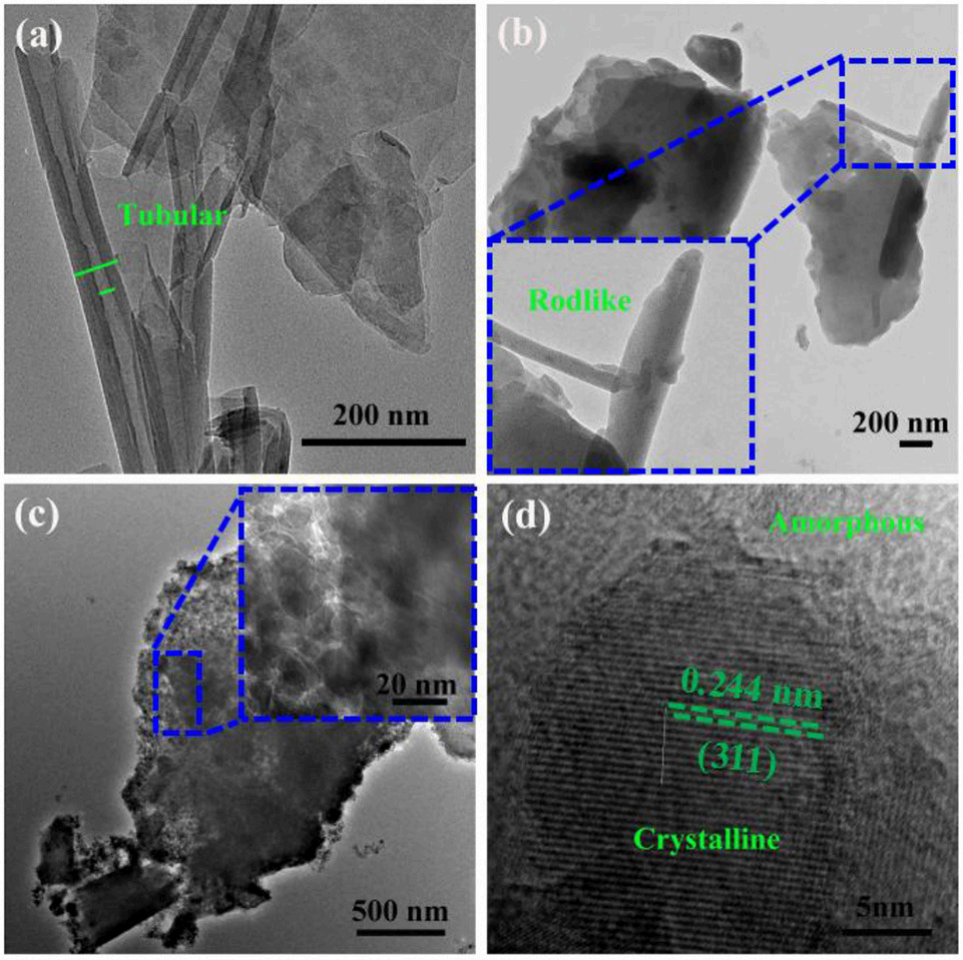
TEM images of **(a)** the raw Kaol, **(b)** Kaol calcined at 1,100°C, **(c)** Kaol-HP, and **(d)** the enlarged electron micrograph of Kaol-HP.

Figure 2a provides the TEM image of Kaol, and it can be found that Kaol is a typical lamellar layered structure with a smooth surface, and some tubular morphology is also observed, which can be attributed to the associated tubular Hal. The length of Hal is around 0.2–2.0 μm while the external and inner diameters are about 50–80 and 20–50 nm, respectively. After being calcined at 1,100°C (Figure 2b), the lamellar morphology of Kaol remains while the tubular structure is transformed into rodlike one, which is possible related to the phase transformation of Hal. After introducing of CoAl_2_O_4_ nanoparticles, the surface of lamellar morphology becomes coarse due to the loading of CoAl_2_O_4_ nanoparticles. The CoAl_2_O_4_ nanoparticles with a diameter of about 10–20 nm are uniformly anchored on the surface of lamellar (Figure 2c). Furthermore, the selected area electron diffraction pattern of Kaol-HP calcined at 1,100°C also confirmed the formation of CoAl_2_O_4_ nanoparticles (Figure S4, see ESI) (Ouahdi et al., 2005; Mindru et al., 2010). In addition, Figure 2d gives an enlarged electron micrograph of Figure 2c, it provides a well-resolved lattice plane with an interplanar spacing of 0.244 nm, corresponding to [311] plane of the cubic *Fd3m* space group, which is identified on the basis of data from the standard CoAl_2_O_4_ database JCPD card no. 10-458 (Kim et al., 2012). The micrograph displays the coexistence of amorphous and crystalline phases, and the crystalline phase might be attributed to the spinel CoAl_2_O_4_ while the amorphous one is related to silicate derived from Kaol (Cho and Kakihana, 1999).

**Figure 3:**
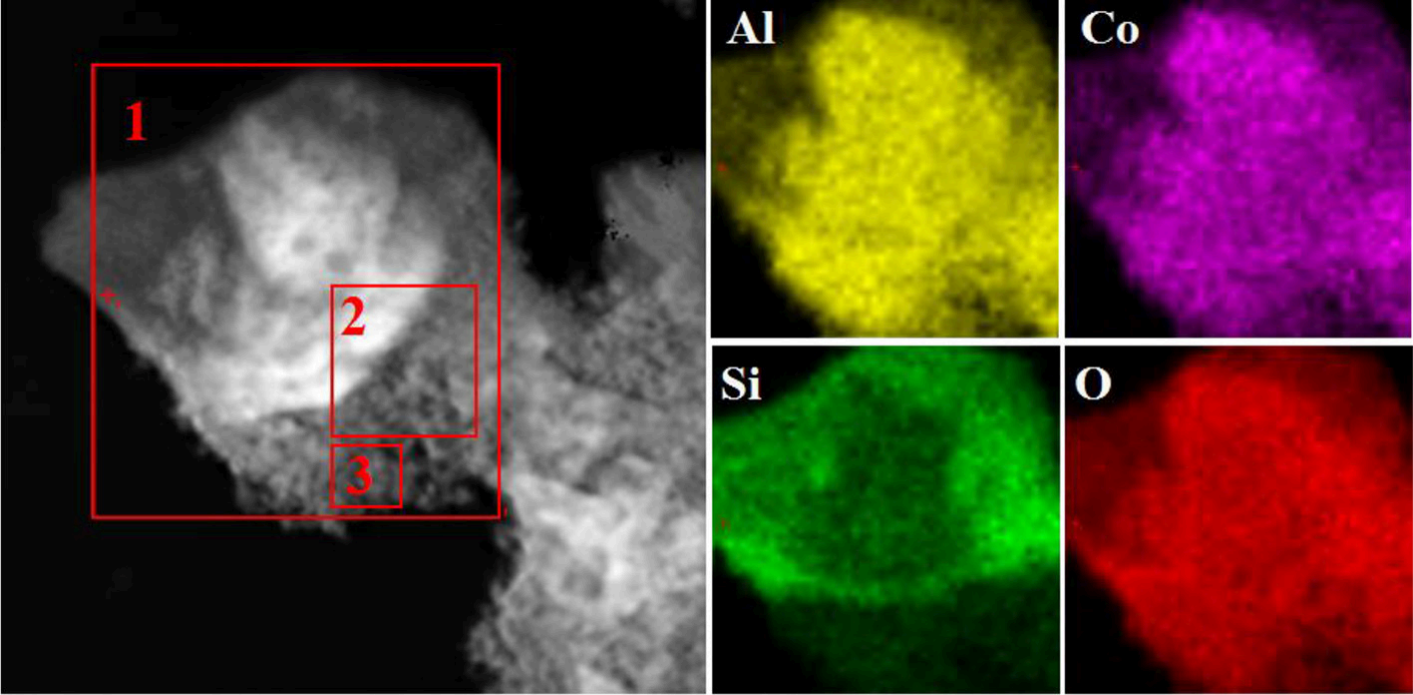
Element mapping of Kaol-HP.

Figure S5 depicts the EDX spectrum of Kaol-HP, and it can be found that Kaol-HP is mainly composed of Co, Al, O, and Si elements. Element mapping of Kaol-HP is illustrated in Figure 3, it is clear that Co element is uniformly distributed on the surface of lamellar, suggesting that the generated CoAl_2_O_4_ is well anchored on the surface of substrate. Furthermore, other elements also present the uniform distribution. By contrast, the edge color of Si element is obscure, which can be attributed to the fact that the edge thickness of the silicate substrate is thin. However, the value of Co/Al decreases from 0.49 to 0.37 to 0.31 with the change of the selected area from boundary to center of Kaol-HP (Table 2), which indicates the loading content of CoAl_2_O_4_ in the edge reign is higher than the center of the silicate substrate. Based on the above analysis, it suggests that Kaol-HP has been successfully prepared.

**Table 2:** The element content of the different selection areas of Kaol-HP.

**Element**	**Atomic (%)**		
	**No. 1**	**No. 2**	**No. 3**
O	67	63.61	51.21
Na	0.95	0.90	0.47
Al	17.01	21.06	31.55
Si	9.49	6.17	1.35
K	0.35	0.22	0
Co	5.18	8.01	15.39
Co/Al	0.31	0.37	0.49
Total	100	100	100

### Effect of different aluminosilicate clay minerals and coloring mechanism

In our previous study (Zhang et al., 2017), it has been confirmed that the introduction of clay minerals, especially aluminosilicate mineral of Hal, was in favor of decreasing the formation temperature of spinel-type CoAl_2_O_4_, and enhancing the color properties of CoAl_2_O_4_ pigment. In order to investigate the effect of the different aluminosilicate minerals on the color properties of hybrid pigments, several of representative aluminosilicate minerals were selected to prepare CoAl_2_O_4_ hybrid pigments including Hal, And, Kaol, Mt, Dic, M47, and M70.

**Figure 4:**
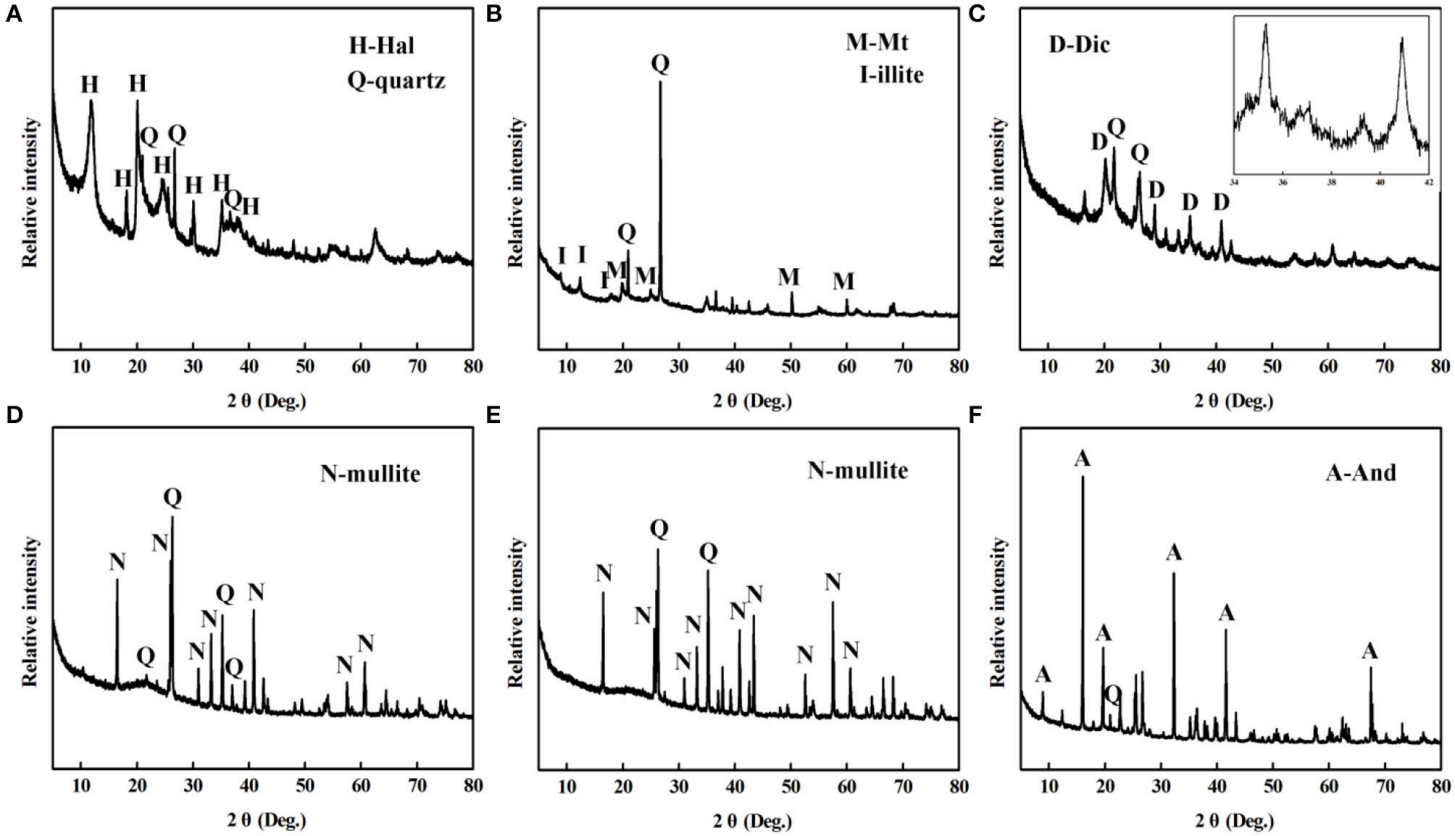
XRD patterns of **(A)** Hal, **(B)** Mt, **(C)** Dic, **(D)** M47, **(E)** M70, and **(F)** And.

The XRD patterns of the different aluminosilicate minerals are provided in Figure 4. Hal is a 1:1 aluminosilicate mineral with the empirical formula Al_2_Si_2_O_5_(OH)_4_, the typical diffraction peaks of Hal are observed at *2*θ = 12.3, 18.3, 20.2, 24.8°, and the diffraction peaks of quartz are located at *2*θ = 20.8, 26.7, and 36.6° (Figure 4A) (Philip et al., 2017). Figure 4B gives XRD pattern of Mt, its characteristic diffraction peaks are located at *2*θ = 5.9, 19.7, and 35.1°, while the diffraction peaks at *2*θ = 12.5 and 17.5° are attributed to illite (Wang et al., 2016; Liang et al., 2017). Dic is a kind of layered silicate mineral, and pertains to 1:1 type of Kaol subgroup. As shown in Figure 4C, the characteristic diffraction peaks between 34 and 39° can be observed, which are assigned to (200), (131), (006), and (133) of Dic, respectively (Zheng et al., 2011). Figures 4D–F depicted the XRD patterns of M47, M70, and And. Mullite is an artificial material, which is synthesized using natural raw materials during 1,100–1,600°C, such as And, kyanite, etc. (Xu et al., 2017; Yuan et al., 2017).

**Figure 5:**
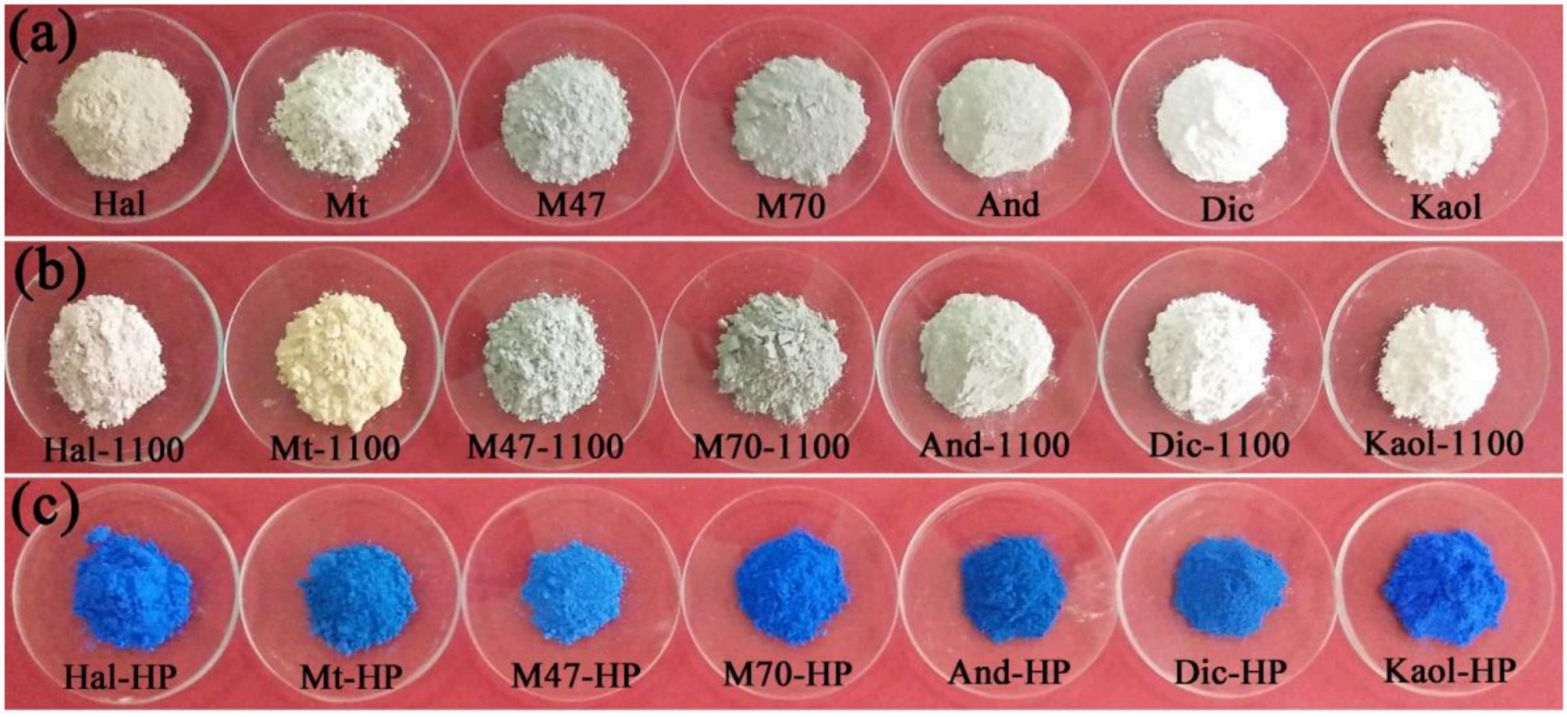
Digital photos of **(a)** different clay minerals, **(b)** the calcined clay minerals at 1,100°C, and **(c)** CoAl_2_O_4_ hybrid pigments derived from different clay minerals, respectively.

Figure 5 shows the digital photos of the raw aluminosilicate clay minerals, the calcined aluminosilicate clay minerals at 1,100°C and CoAl_2_O_4_ hybrid pigments derived from different aluminosilicate clay minerals, respectively. It is obvious that different clay minerals present different colors. Dic and Kaol are white, Hal and Mt are light brown, while M47, M70, and And are gray. After being calcined at 1,100°C (Figure 5b), Kaol and Dic are white, Mt is yellowish-brown, Hal was light pink, and the others (M47, M70, and And) are gray. The digital photos the as-prepared hybrid pigments are exhibited in Figure 5c, it is found that all hybrid pigments present typical blue, but there are difference among different hybrid pigments. By contrast, the color of Kaol-HP is optimal, which is also consistent with their color parameters (Table 3). Kaol-HP exhibits the maximum b^*^ and C^*^ values, followed by M70-HP, M47-HP, Hal-HP, respectively, while Mt-HP and And-HP indicate the worst lightness (L^*^). It suggests that the colors of the hybrid pigments might be related to the types and compositions of aluminosilicate clay minerals.

**Table 3:** CIE parameters of the different CoAl_2_O_4_ hybrid pigment.

**Hybrid pigments**	L^*^	a^*^	b^*^	C^*^
Hal-HP	54.60	−4.30	−50.10	50.28
Kaol-HP	48.11	2.64	−63.75	63.80
Mt-HP	29.66	−13.97	−43.33	45.53
Dic-HP	49.14	−8.27	−47.63	48.34
And-HP	30.26	−10.41	−54.69	55.67
M47-HP	40.69	−14.65	−52.43	54.44
M70-HP	33.53	−1.63	−58.45	58.47

**Figure 6:**
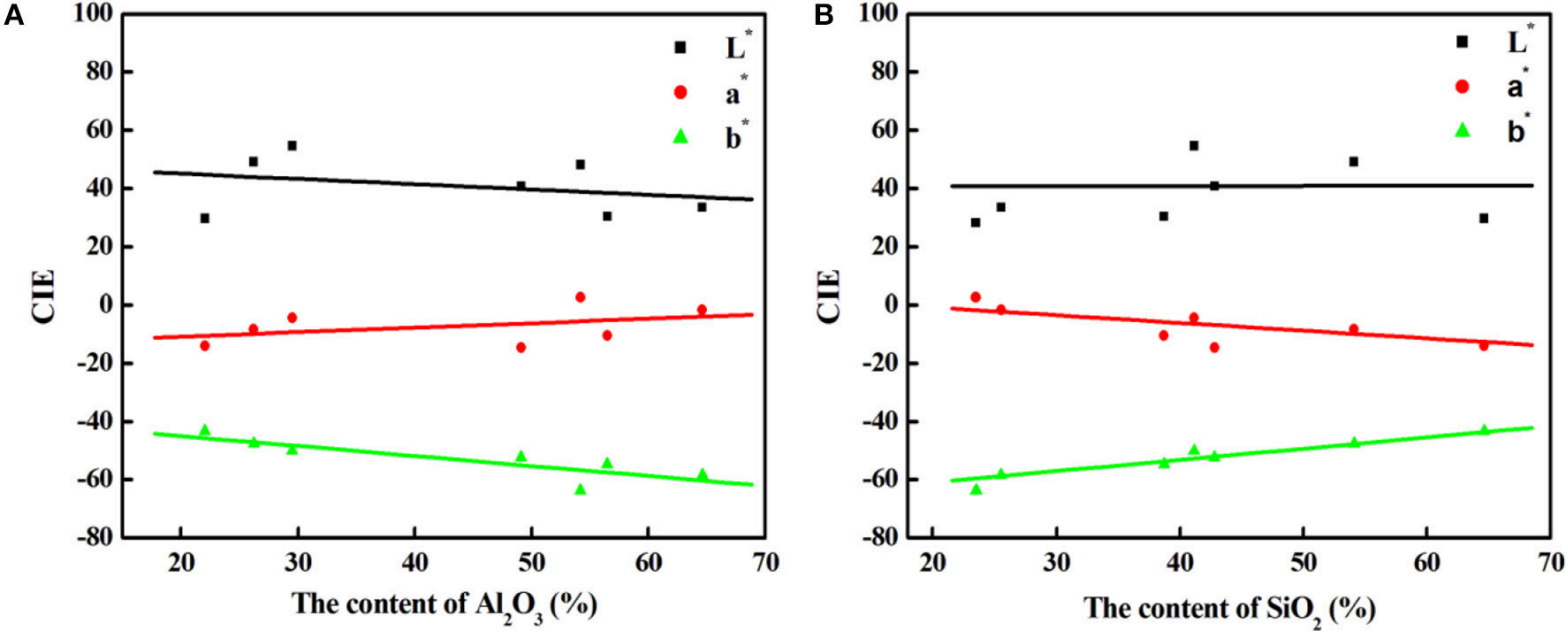
The relationship between the CIE parameters of CoAl_2_O_4_ hybrid pigments and the content of Al_2_O_3_ **(A)** and SiO_2_ **(B)** of different clay minerals, respectively.

**Figure 7:**
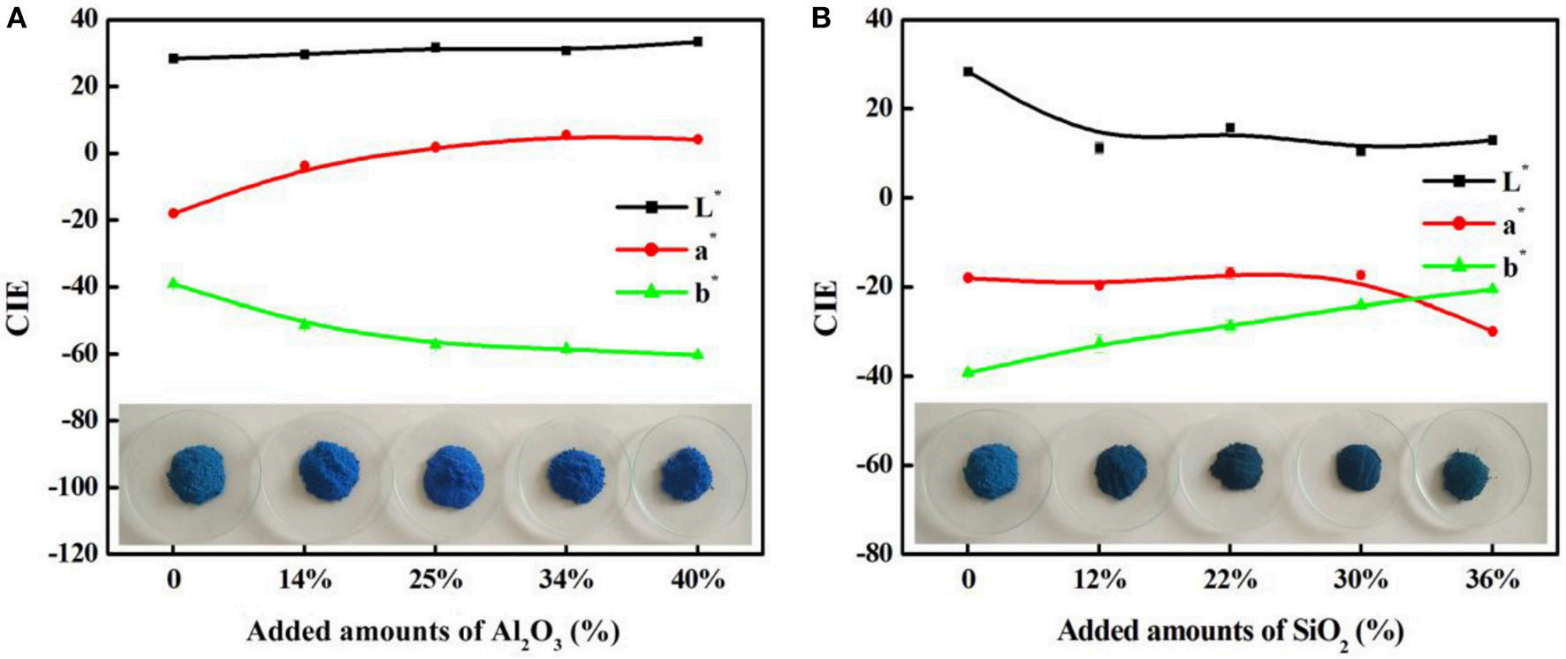
The relationship between the CIE parameters of CoAl_2_O_4_ hybrid pigments prepared after incorporating of different added amounts of **(A)** Al_2_O_3_ and **(B)** SiO_2_ without clay minerals, respectively.

As shown in Table 1, the main compositions of the involved aluminosilicate clay minerals are Al_2_O_3_ and SiO_2_. Therefore, the relationship between the content of Al_2_O_3_ or SiO_2_ and the color parameters of hybrid pigments are investigated, and Figure 6 gives the relationship between the CIE parameters of CoAl_2_O_4_ hybrid pigments and the content of Al_2_O_3_ and SiO_2_ of different aluminosilicate clay minerals, while Table S2 summarizes the content of Al_2_O_3_ and SiO_2_ of the clay minerals and the CIE of hybrid pigments. As a whole, the value of a^*^ of hybrid pigments increases with the increase in the content of Al_2_O_3_, but the b^*^ value becomes more negative. It indicates the blue color of hybrid pigments become deeper with the increase in the Al_2_O_3_ content. However, the b^*^ value is more positive with the increase in the content of SiO_2_, indicating a poor blue color. Therefore, Al_2_O_3_ and SiO_2_ of aluminosilicate clay minerals plays an important role in adjusting the color of CoAl_2_O_4_ hybrid pigments, but the ultimate color properties of hybrid pigments results from the synergy of various compositions of aluminosilicate clay minerals, besides their colors. In order to prove above proposal, Al_2_O_3_ and SiO_2_ with different added amounts were employed to fabricate hybrid pigments without clay minerals, respectively. As shown in Figure 7, the b^*^ value of hybrid pigments prepared using Al_2_O_3_ gradually decreases with the increase in the added amounts of Al_2_O_3_ (more negative). On the contrary, the b^*^ value of hybrid pigments derived from SiO_2_ increases with the increase in the amount of SiO_2_ (more positive), which might be attributed to the formation of cobalt silicate (Llusar et al., 2001). This variation trend is also agreement with the results of Figures 6A,B.

**Figure 8:**
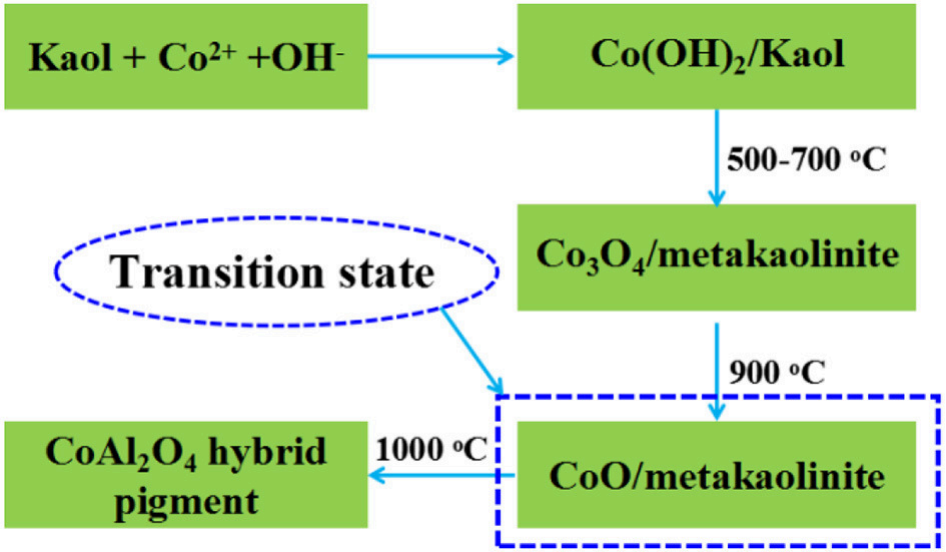
Possible formation process of CoAl_2_O_4_ hybrid pigment prepared using Kaol and Co^2+^ without Al^3+^.

In addition, the pH value of the reaction system is crucial to cobalt-aluminum double hydroxides (Co/Al DH) (Zhang et al., 2017), the excess OH^−^ affects the molar ratio of Co/Al in the ultimate CoAl_2_O_4_ due to the dissolution loss of Al in alkaline medium, which directly determinate the color properties of pigments. In order to prove this effect, HCl was added into the centrifugate after the co-precipitation reaction, it can clearly observe the white precipitate upon the addition of HCl (1.0 M), and the precipitate is confirmed to be Al(OH)_3_ using FTIR technique (Figure S6, see ESI) (Kamaraj and Vasudevan, 2016). In addition, the higher the pH values of the reaction, the more the contents of white precipitate (Figure S6, see ESI). When the pH value of reaction system is above 10, the generated Al(OH)_3_ will partially dissolve and the molar ratio of Co^2+^/Al^3+^ is <2, which can be confirmed by the EDX of CoAl_2_O_4_ in the absence clay minerals during preparation. As shown in Figure S7, the value of Co/Al of CoAl_2_O_4_ in the absence of clay minerals is about 0.62, and thus the Al loss is about 20% due to the dissolution of Al(OH)_3_ at alkaline medium during preparation of precursor. Therefore, the incorporation of aluminosilicate clay mineral may be an ideal natural aluminum sources to compensate the aluminum loss due to the dissolution of Al(OH)_3_ at alkaline medium during preparation of precursor, keeping an optimum molar ratio of Co^2+^/Al^3+^ for formation of spinel CoAl_2_O_4_ pigment. It can be inferred that the Al originated from clay minerals about 20% might be participated in reaction and enter into the octahedral positions of CoAl_2_O_4_ spinel structure to form CoAl_2_O_4_-silicate solid solution (Tang et al., 2018). This is also can be confirmed by the production derived from Co^2+^ salt and Kaol in the absence of Al^3+^ salt, which is prepared using the same procedure with Kaol-HP. As shown in Figure S8, the XRD pattern of the product presents the characteristic diffraction peaks of CoAl_2_O_4_ suggesting that Kaol can be served as an aluminum source to form CoAl_2_O_4_. The evolution process might be depicted by the following process (Figure 8) (Zhong et al., 1999; Cava et al., 2006).

### Formation mechanism of CoAl_2_O_4_ hybrid pigments

**Scheme 1:**
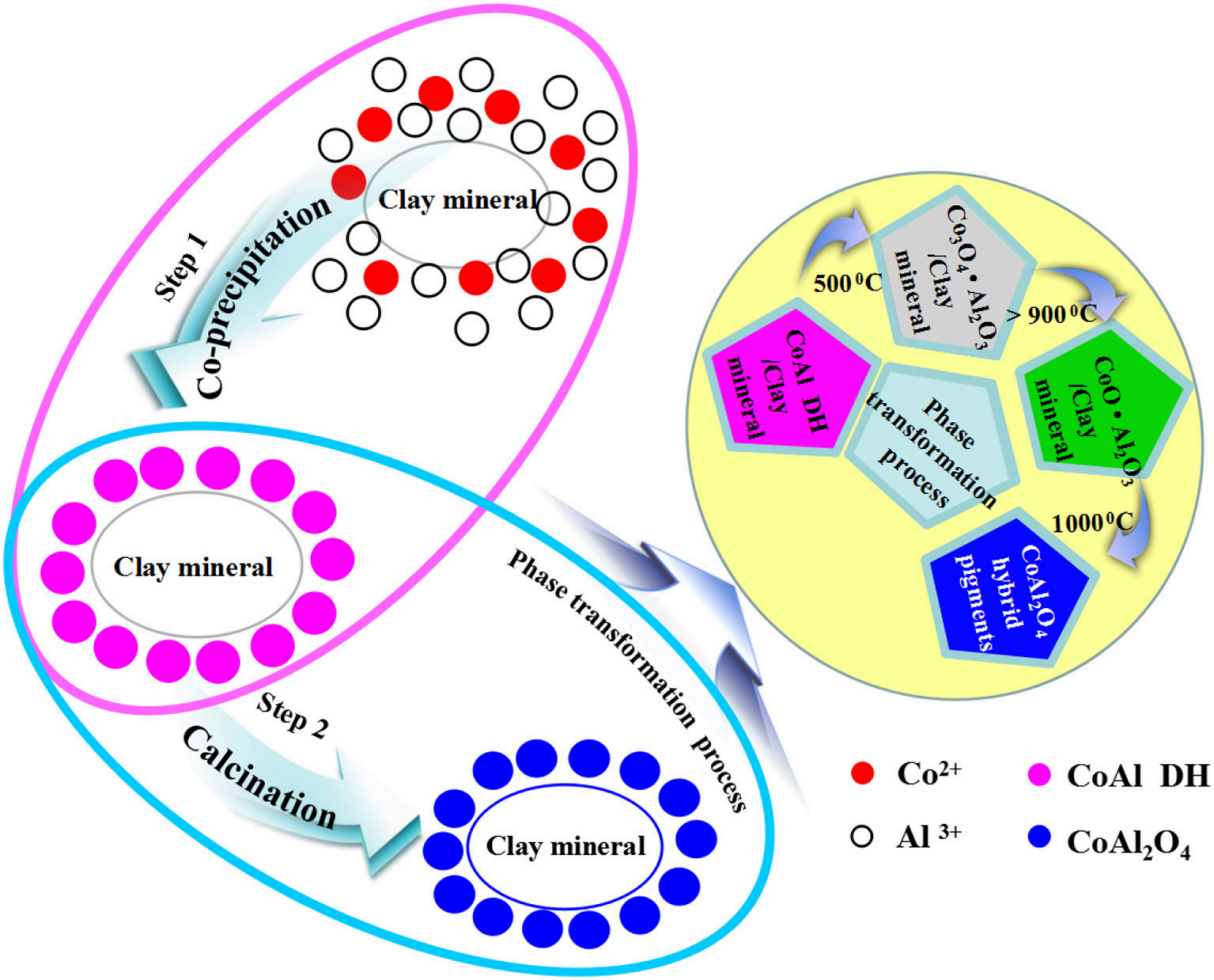
Schematic for Kaol-HP formation process and the relevant phase transformation process during calcining.

Based on the above results, the possible formation mechanism of CoAl_2_O_4_ hybrid pigments was proposed (Scheme 1). During the co-precipitation reaction, the Co^2+^ and Al^3+^ were firstly adsorbed on the surface of clay minerals due to the electrostatic interaction and ion exchange between metal ions and clay minerals, and then Co^2+^ and Al^3+^ were transformed into the hydroxides and deposited on the surface of clay mineral after introducing of OH^−^. Due to the difference in the solubility constant of Al(OH)_3_ and Co(OH)_2_, Al^3+^ was firstly began to precipitate at lower pH, and then Co^2+^ was transformed into Co(OH)_2_ at higher pH (Loganathan et al., 1977; He and Becker, 1997; Matjie et al., 2003; Akdemir et al., 2011). When the pH of reaction system was above 10, Co^2+^ almost precipitated completely, but Al(OH)_3_ partially dissolved to form ${\text{AlO}}_{2}^{-}$. As a result, the molar ratio of Co^2+^/Al^3+^ is <2. (Lavrenčič Štangar et al., 2003; Carta et al., 2005; Xu et al., 2008; Tielens et al., 2009; Kurajica et al., 2012). Therefore, CoAl DH was decomposed to form amorphous Co_3_O_4_ phase at around 400–500°C accompanied with the presence of highly amorphous Al_2_O_3_ once heating. With the increase in the calcining temperature (500–700°C), the amorphous Co_3_O_4_ was transformed into the spinel-type Co_3_O_4_ while Kaol was turned into metakaolinite (2SiO_2_·Al_2_O_3_) (Yu et al., 2009; Duan et al., 2011), and Co_3_O_4_ was then progressively transformed into CoAl_2_O_4_ phase above 900°C, thus the content of spinel CoAl_2_O_4_ phase gradually increased with the vanishing of Co_3_O_4_ phase as the calcining temperature was above 1,000°C (Zayat and Levy, 2000; Duan et al., 2011; Álvarez-Docio et al., 2017; Zhang et al., 2018). In addition, metakaolinite was firstly transformed into spinel SiAl_2_O_5_ and amorphous SiO_2_, and then the spinel SiAl_2_O_5_ changed into amorphous SiO_2_ and low-order crystalline α-Al_2_O_3_ above 1,000°C (Chen et al., 2000; Ribeiro et al., 2005; Veselý et al., 2010; Wu et al., 2017). In fact, the thermal reduction of Co^3+^ to Co^2+^ was simultaneous with the diffusion and reorganization of Co^2+^ and Al^3+^ ions derived from the precursor and clay minerals during this process.

It is well-known that the mass transfer process was the controlling rate step during CoAl_2_O_4_ preparation (Gabrovska et al., 2014). Therefore, the aluminosilicate clay minerals induced the anchoring of CoAl DH on their surface preventing from the free aggregations of CoAl DH, which could effectively decrease mass transfer resistance to reduce the time and calcining temperature for formation of spinel CoAl_2_O_4_. In addition, it was in favor of controlling the size and particle size distribution of CoAl_2_O_4_ nanoparticles during calcinaion process. What's more, Al^3+^ derived from aluminosilicate clay mineral also participated in the CoAl_2_O_4_ crystallization by the diffusion from substrate to compensate the aluminum loss during the co-precipitation reaction (Wang et al., 2006; Álvarez-Docio et al., 2017). Therefore, it may explain why the bright blue CoAl_2_O_4_ hybrid pigments could be obtained at 1,100°C for 2 h, but the traditional solid phase method for preparation of CoAl_2_O_4_ must be conducted at high temperature (>1,200°C) for a long time (Ji et al., 2000; Lorite et al., 2012). Especially, the hybrid pigments derived from the 1:1 style aluminosilicate clay minerals presented high CIE parameters. Due to the higher affinity of CoO to Al_2_O_3_ than SiO_2_, and the CoO was easily arrived to the interphase of α-Al_2_O_3_ originating from aluminosilicate clay mineral to generate CoAl_2_O_4_ (Ahmed et al., 2012). For 2:1 style aluminosilicate clay minerals, the aluminosilicate clay minerals transformed into amorphous SiO_2_ and α-Al_2_O_3_ at high temperature, and the content of SiO_2_ is much higher than that of α-Al_2_O_3_. CoO might react with partially SiO_2_ to form the CoSiO_3_, which led to poor color properties of hybrid pigment. In order to prove above proposal, XRD patterns of Kaol-HP and Mt-HP are compared (Figure S9), it is clear that the CoSiO_3_ (JCPD card no. 72-1508) occurs in Mt-HP accompanied with SiO_2_ (JCPD card no. 86-0680), but they cannot be found in XRD pattern of Kaol-HP.

## Conclusions

In summary, different aluminosilicate clay minerals were employed to prepare the CoAl_2_O_4_ hybrid pigment with different color properties. It was found that the more content of Al_2_O_3_, the better color properties of hybrid pigment (higher lightness and blue), suggesting that the rich-aluminum silicate mineral was appropriate for preparation of bright blue cobalt blue pigment. The aluminosilicate clay mineral was served as a carrier to load CoAl_2_O_4_ nanoparticles, preventing from the aggregation and controlling the size of CoAl_2_O_4_ nanoparticles during calcining process. What's more, it was an ideal natural aluminum sources to compensate the aluminum loss due to the dissolution of Al(OH)_3_ at alkaline medium during preparation of precursor, keeping an optimum molar ratio of Co^2+^/Al^3+^ for formation of spinel CoAl_2_O_4_ pigments during high-temperature crystallization. Therefore, this study may provide a feasible strategy not only to develop low-cost and bright blue cobalt blue pigments, but also to control the color properties of cobalt blue by adjusting the types of clay minerals to meet the personalized demand in the practice application.

## Author contributions

AZ and XW contribute to the experiment process, data analysis, and paper preparation. BM and AW are mainly responsible for the design of experiment, data analysis and paper revision. LW contributes to the samples characterization.

### Conflict of interest statement

The authors declare that the research was conducted in the absence of any commercial or financial relationships that could be construed as a potential conflict of interest. The reviewer SW and handling Editor declared their shared affiliation.
