# Supplementary material for: Formation and Coloring Mechanism of Typical Aluminosilicate Clay Minerals for CoAl2O4 Hybrid Pigment Preparation
Source: Front Chem. 2018 Apr 19;6:125. doi: 10.3389/fchem.2018.00125 (PMC5917090; doi:10.3389/fchem.2018.00125)
Supplement: Supplementary file 1 [file Presentation1.pdf]

Formation and coloring mechanism of typical aluminosilicate clay minerals for $CoAl_2O_4$ hybrid pigment preparation

Anjie Zhang[a,b,c], Bin Mu[a*], Xiaowen Wang[a, b], Lixin Wen[c], Aiqin Wang[a*]

[a] Key Laboratory of Clay Mineral Applied Research of Gansu Province, Center of Eco-material and Green Chemistry, Lanzhou Institute of Chemical Physics, Chinese Academy of Sciences, Lanzhou 730000, P.R. China

[b] University of Chinese Academy of Sciences, Beijing 100049, P.R. China

[c] Northwest Yongxin coatings Limited company, Lanzhou 730046, P.R. China

[*] Corresponding authors. E-mail addresses: mubin@licp.cas.cn (B. Mu) and aqwang@licp.cas.cn (A.Q. Wang); Fax: +86 931 8277088; Tel: +86 931 4868118.

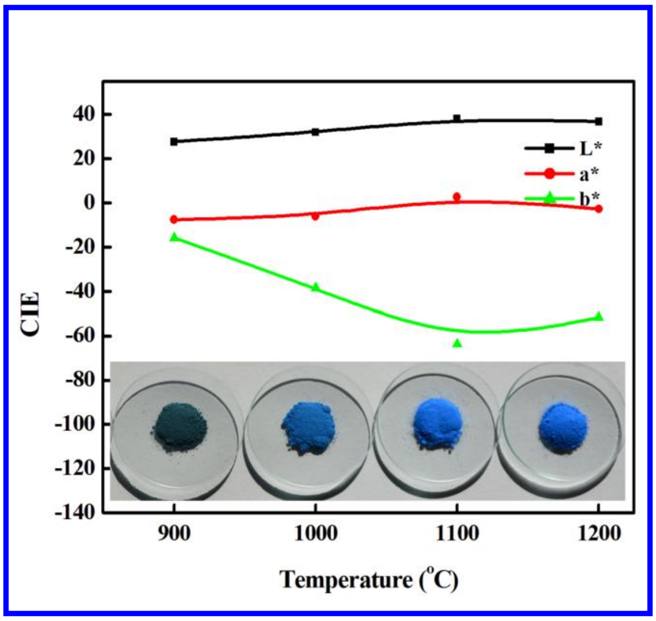

Fig. S1 CIE parameters and photographs of Kaol-HP prepared using at different temperatures.

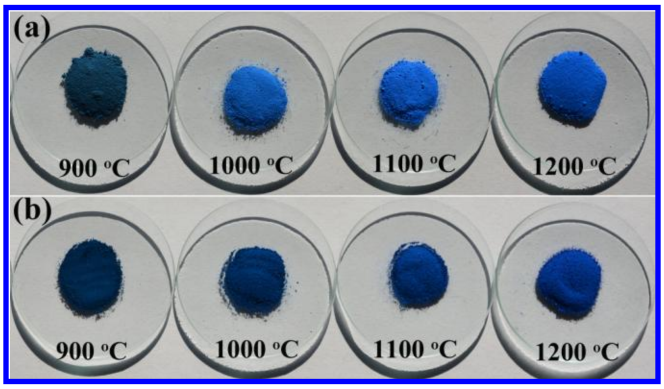

Fig. S2 Digital photographs of (a) Kaol-HP (b) $CoAl_2O_4$ calcined at different temperatures.

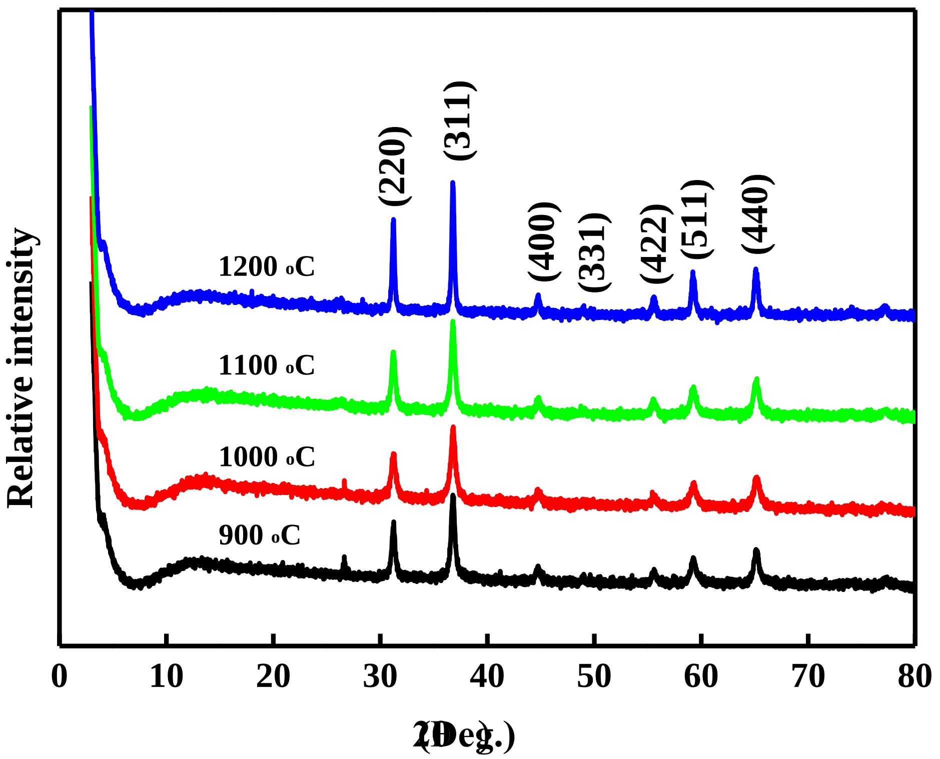

Fig. S3 XRD patterns of the Kaol hybrid pigments calcined at different temperatures.

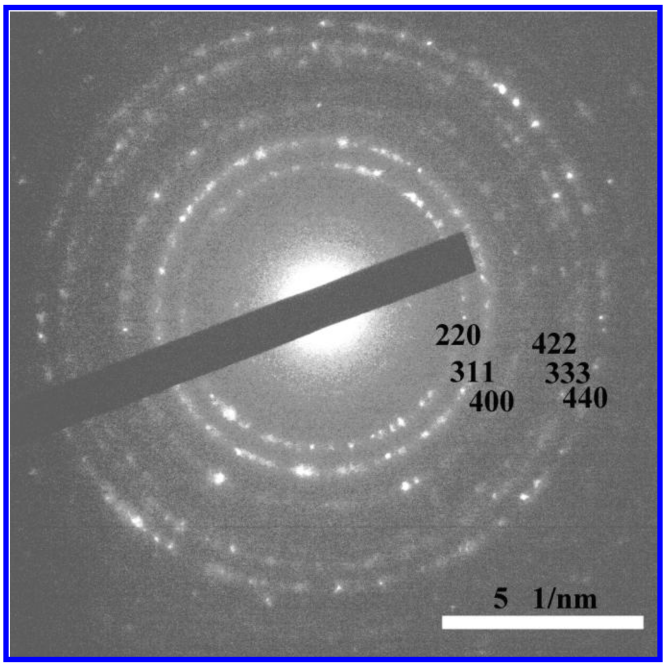

Fig. S4 District electron diffraction spectrum of Kaol-HP.

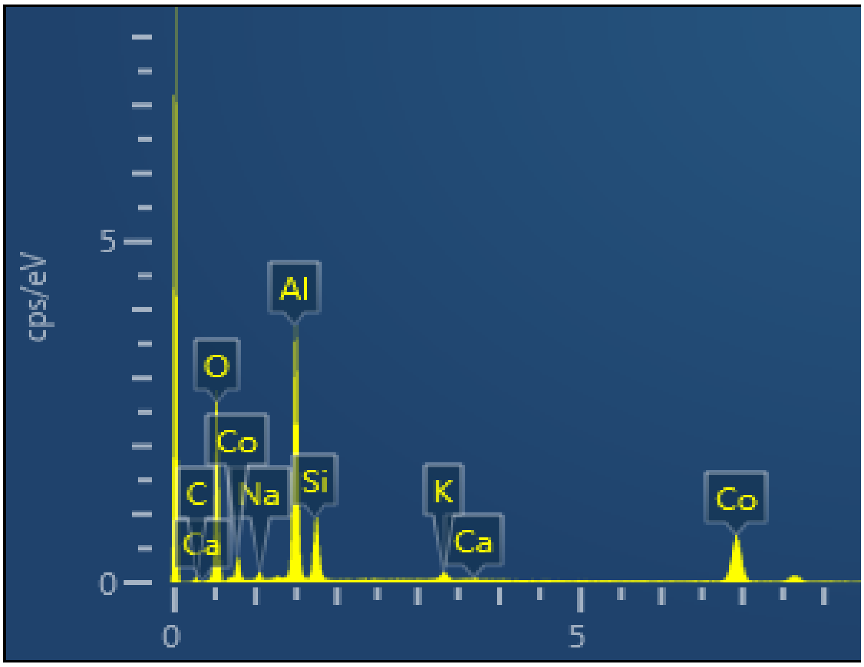

Fig. S5 EDX spectrum of Kaol-HP.

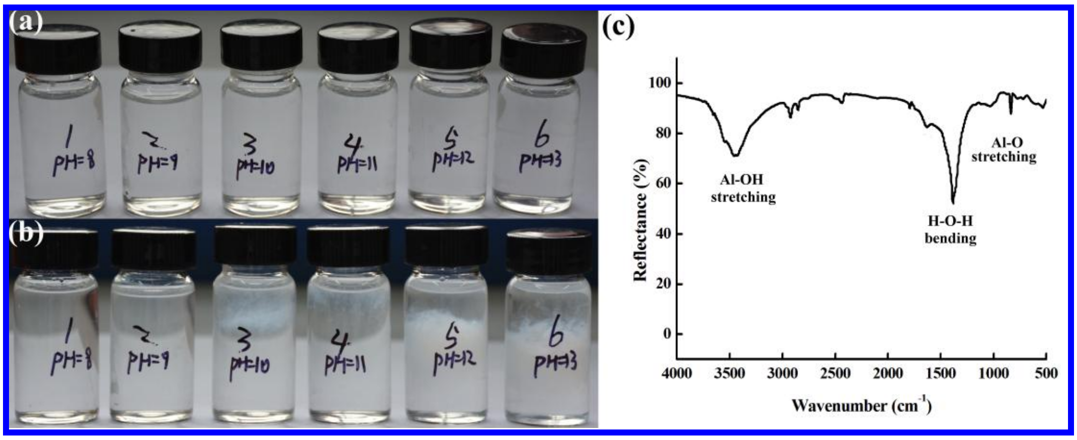

Fig. S6 (a and b) Digital photos of the centrifugate before and after adding of HCl, and (c) FTIR spectrum of the above white precipitate.

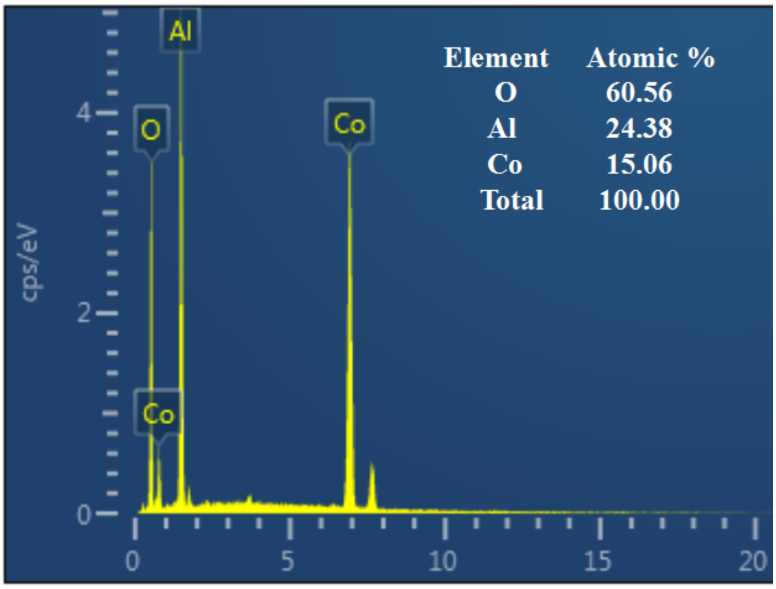

Fig. S7 EDX curves of $CoAl_2O_4$ calcined at 1200 ℃ in the absence of clay minerals.

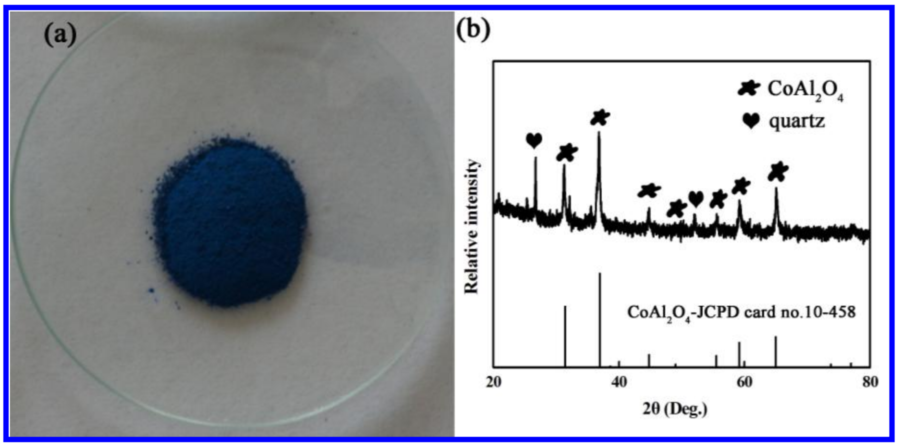

Fig. S8 (a) Digital photo and (b) XRD pattern of the as-prepared sample using $Co(NO_3)_2{\cdot}6H_2O$ and Kaol in the absence of $Al(NO_3)_3{\cdot}9H_2O$ at the same conditions.

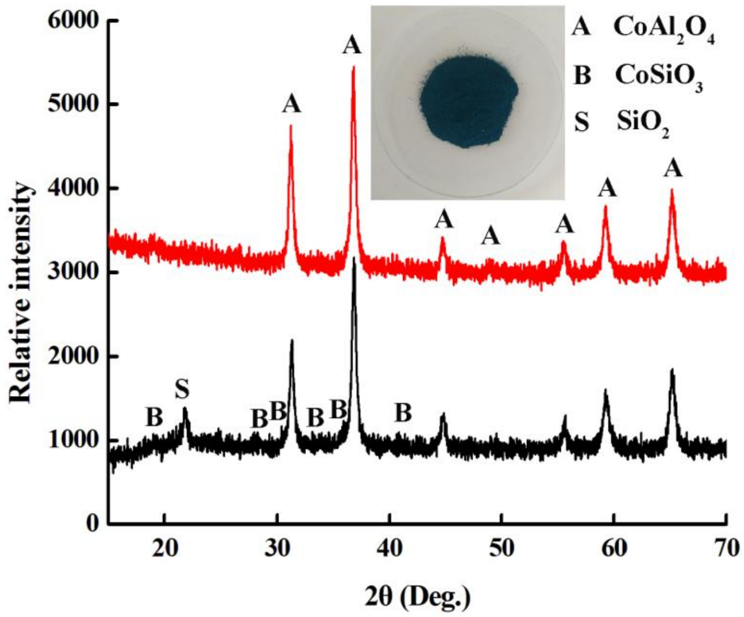

Fig. S9 XRD pattern of the Mt-HP (black line) and Kaol-HP (red line) (The insert is the photo of the as-prepared $CoSiO_3$).

Table S1 Color parameters, crystallite sizes and cell parameters of $CoAl_2O_4$ pigments and $CoAl_2O_4$ hybrid pigments.

[a]Samples	L^*	a^*	b^*	Crystallite sizes (nm)
$CoAl_2O_4$-900	29.8	-4.5	-25.0	-
Kaol-HP-900	27.6	-7.6	-15.9	-
$CoAl_2O_4$-1000	30.2	-3.8	-27.6	28
Kaol-HP-1000	31.9	-6.1	-38.6	20
$CoAl_2O_4$-1100	31.6	-22.5	-27.8	37
Kaol-HP-1100	38.11	2.64	-63.8	22
$CoAl_2O_4$-1200	31.8	-14.8	-29.1	47
Kaol-HP-1200	36.7	-2.8	-51.8	25

[a] The number in the column of samples represents the corresponding calcining temperatures .

Table S2 Chemical composition of different clay minerals after acid treatment and the CIE parameters of the different $CoAl_2O_4$ hybrid pigment derived from different clay minerals.

Clay minerals	Al_2O_3 (%)	SiO_2 (%)	Hybrid pigments	L^*	a^*	b^*	C^*
Hal	29.49	41.15	Hal-HP	54.6	-4.3	-50.1	50.28
Kaol	54.2	23.5	Kaol-HP	48.11	2.64	-63.75	63.80
Mt	22	64.6	Mt-HP	29.66	-13.97	-43.33	45.53
Dic	26.2	54.1	Dic-HP	49.14	-8.27	-47.63	48.34
And	56.5	38.7	And-HP	30.26	-10.41	-54.69	55.67
M47	49.1	42.8	M47-HP	40.69	-14.65	-52.43	54.44
M70	64.6	25.5	M70-HP	33.53	-1.63	-58.45	58.47
